# A genome-wide 20 K citrus microarray for gene expression analysis

**DOI:** 10.1186/1471-2164-9-318

**Published:** 2008-07-03

**Authors:** M Angeles Martinez-Godoy, Nuria Mauri, Jose Juarez, M Carmen Marques, Julia Santiago, Javier Forment, Jose Gadea

**Affiliations:** 1 Instituto de Biología Molecular y Celular de Plantas (IBMCP), Laboratorio de Genomica (Universidad Politécnica de Valencia – Consejo Superior de Investigaciones Científicas), Avenida de los Naranjos s/n, E46022 Valencia, Spain; 2 Instituto Valenciano de Investigaciones Agrarias (IVIA), Carretera Moncada-Náquera, Km.4.5, E46113 Moncada, Valencia, Spain

## Abstract

**Background:**

Understanding of genetic elements that contribute to key aspects of citrus biology will impact future improvements in this economically important crop. Global gene expression analysis demands microarray platforms with a high genome coverage. In the last years, genome-wide EST collections have been generated in citrus, opening the possibility to create new tools for functional genomics in this crop plant.

**Results:**

We have designed and constructed a publicly available genome-wide cDNA microarray that include 21,081 putative unigenes of citrus. As a functional companion to the microarray, a web-browsable database [1] was created and populated with information about the unigenes represented in the microarray, including cDNA libraries, isolated clones, raw and processed nucleotide and protein sequences, and results of all the structural and functional annotation of the unigenes, like general description, BLAST hits, putative Arabidopsis orthologs, microsatellites, putative SNPs, GO classification and PFAM domains. We have performed a Gene Ontology comparison with the full set of Arabidopsis proteins to estimate the genome coverage of the microarray. We have also performed microarray hybridizations to check its usability.

**Conclusion:**

This new cDNA microarray replaces the first 7K microarray generated two years ago and allows gene expression analysis at a more global scale. We have followed a rational design to minimize cross-hybridization while maintaining its utility for different citrus species. Furthermore, we also provide access to a website with full structural and functional annotation of the unigenes represented in the microarray, along with the ability to use this site to directly perform gene expression analysis using standard tools at different publicly available servers. Furthermore, we show how this microarray offers a good representation of the citrus genome and present the usefulness of this genomic tool for global studies in citrus by using it to catalogue genes expressed in citrus globular embryos.

## Background

In the last years, microarray technology has demonstrated the power of the high-throughput study of gene expression in the unravelling of key processes of plant biology [2-4]. Microarrays have become especially relevant for crop species where little genome information is available, and where intensive laboratory work is necessary to get insight into a particular biological process, as well as to identify candidate target genes for future breeding [5].

Citrus is the most economically important fruit crop in the world, with a total production of 105 million metric tons. There is a plethora of important commercial species and varieties, including sweet oranges, mandarins, lemons and grapefruits. Variety improvement efforts have been hampered by general characteristics of citrus biology, such as apomixis, sexual incompatibility or prolonged juvenility, that limit classical molecular biology approaches. Functional genomics is then viewed as a relatively easy way to move forward into the identification of candidate genes of agronomical relevance, and to the understanding of biological processes important for citriculture.

Two years ago, aiming to develop genomic tools to assist future citrus research, we generated an EST collection covering a wide range of tissues and developmental stages, as well as biotic and abiotic stress situations, and constructed a first-generation cDNA microarray containing 6875 putative unigenes to initiate the characterization of citrus transcriptome [6]. This first microarray has been used so far to monitor the transcriptional response of citrus in ovaries and young fruit during development and ripening of citrus flesh [7], during CTV virus infection [8], or under water stress conditions [9], as well as to predict citrus varieties using expression profiles [10].

However, to perform expression analysis in citrus at a more global scale, new microarray platforms with increased genome representation are mandatory. cDNA microarrays are still a valuable tool for transcriptomic analysis in many species [11-14]. In plants, a cDNA array containing more than 10.000 unigenes has been recently generated for canola [15]. Although cDNA microarrays are being gradually substituted by oligo arrays due to reduction of manipulation steps during fabrication, and to their ability to detect similar members of some gene families, the validity of both platforms to perform reproducible and biologically consistent results has been clearly demonstrated, and the lack of concordance between microarray platforms has proven to be a failure of the metrics used to evaluate such concordance [16]. Moreover, cDNA microarrays seems to be the best option for comparative, evolutionary and ecological studies of closely related species [17], taking profit that cross-hybridization is expected to occur in cDNA arrays when sequence homology between targets and probes is higher than 70% [18]. This is especially relevant for citrus, a tree grown as a combination of the fruit-producing scion variety bud-grafted onto a rootstock variety adapted to the soil and environment, as many studies combine both parts of the tree. Here we describe the design and creation of a publicly available cDNA microarray that include 21,081 putative unigenes of citrus. Our microarray complements the recently released Citrus Affymetrix GeneChip [19] and provides an alternative tool to perform global transcriptomic assays in these species. Although the majority of gene fragments spotted on the array were isolated from *Citrus clementina*, the cDNA nature of our microarray extends its use to any citrus species [8,10], allowing also comparison of scion/rootstock expression [9]. To illustrate their utility, we use this microarray to catalogue genes expressed in citrus globular embryos, and show how embryogenesis in citrus proceeds expressing a similar set of genes as it does in Arabidopsis.

## Results and Discussion

### Microarray design

**Figure 1:**
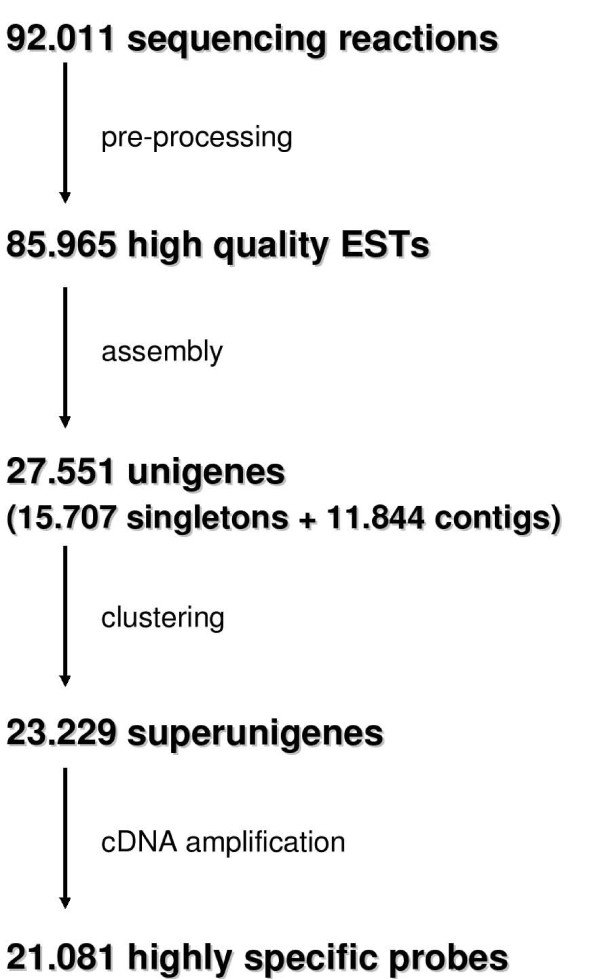
**Microarray design**. ESTs were pre-processed and assembled to obtain the non-redundant unigene set, and unigenes were further clustered in 'supercontigs' grouping unigenes with extensive sequence overlapping.

The starting material for the selection of probes to be printed in the microarray were the cDNA clone collection generated by the Citrus Functional Genomics Project (CFGP) [6], and a number of external clones integrated in this collection [20,21], as well as the 92,011 trace files generated from all of them. Details about the source cDNA libraries can be found at CFGP homepage [1]. Figure 1 shows the steps in the selection of citrus unigenes to be represented in the microarray. After vector and low quality sequence trimming of the raw sequences obtained from the 92,011 chromatograms available, 85,965 high quality ESTs were obtained, with an average length of 710 bases. Following sequence assembly of this EST dataset, 15,707 singletons were identified and the remaining ESTs clustered into 11,844 contigs (27,551 unigenes total).

To further reduce the sequence redundancy in the 27,551 citrus unigenes, a number of unigene clusters (or "superunigenes"), grouping different unigenes with extensive sequence overlapping, was obtained (see Material and Methods). Members of a superunigene could represent highly similar family members, alternative splicing or polymorphisms. Since their sequence is very similar, they are expected to identify the same mRNA species under standard hybridization conditions if used in cDNA microarrays [18]. In an attempt to reduce such eventual spot cross-hybridizations, only one representative cDNA clone per superunigene was selected to be printed in the microarray, and only clones producing a single PCR product were accepted (see Material and Methods), which produced a total of 21,081 reasonably specific cDNA probes. Additional file 1 shows functional annotation of the genes represented in the microarray, including the ID, description and E value of the first BLAST hit from the databases used for annotation (UniRef90 [22] and Arabidopsis TAIR full set of proteins [23]), as well as their Gene Ontology classification [24] and pfam domains [25].

### Microarray representation

In order to estimate the genomic representation of the microarray, Arabidopsis sequences similar to the citrus unigenes present in the microarray were identified and used for Gene Ontology [24] functional classification (see Material and Methods). Arabidopsis similar sequences (BLASTX E value lower than 10^-20^) were found for 13,266 citrus unigenes (63% of the total unigenes in the microarray). The remaining 37% did not have any match in the Arabidopsis genome with a BLASTX E value lower than 10^-20^. As discussed in a former paper [6] a proportion of these could be citrus or tree-specific genes, and demonstrate the importance of molecular studies in crop species, that can reveal interesting proteins and new biosynthetic pathways not yet discovered in other systems.

Table 1 shows the similarity between distribution of citrus unigenes in the microarray and Arabidopsis similar sequences along the main GO functional categories in the "Biological Process" ontology (the total distribution of citrus and Arabidopsis genes along the different GO functional categories is shown in Additional file 2). An overview of selected functional categories shows that the microarray includes broad representation of genes involved in many categories covering virtually every aspect of plant biology. For example, 663 genes involved in aminoacid metabolism (583 for Arabidopsis), 140 genes involved in photosynthesis (144 for Arabidopsis), or 818 in signal transduction (997 for Arabidopsis), as well as 461 involved in secondary metabolic processes (421 for Arabidopsis) and 1352 genes involved in response to stress (1094 for Arabidopsis) are represented in the array. These results indicate that the citrus microarray offers a good representation of the citrus genome and show the usefulness of this genomic tool for global studies in citrus. However, we could not find citrus similar sequences for around 50% of Arabidopsis genes. Although some of them do not necessarily have to match a corresponding ortholog in citrus, it is reasonable to think that this is the case for many of them, meaning that still more effort will be necessary to generate a whole-genome citrus microarray.

**Table 1:** Genome-wide feature of the microarray. Comparison of numbers and percentages of genes at the Biological Process Gene Ontology between citrus and Arabidopsis.

Genome-wide feature of the microarray		
	Citrus (%)	Arabidopsis (%)

anatomical structure morphogenesis	569 (2.7)	478 (1.79)
amino acid and derivative metabolic process	663 (3.15)	583 (2.19)
signal transduction	818 (3.89)	997 (3.74)
cell cycle	170 (0.81)	212 (0.8)
cell differentiation	417 (1.98)	345 (1.3)
cellular homeostasis	147 (0.7)	168 (0.63)
DNA metabolic process	281 (1.33)	474 (1.78)
transcription	891 (4.23)	1919 (7.21)
protein modification process	940 (4.46)	1563 (5.87)
translation	594 (2.82)	1392 (5.23)
death	167 (0.79)	116 (0.44)
growth	318 (1.51)	318 (1.51)
biosynthetic process	1942 (9.22)	2947 (11.07)
carbohydrate metabolic process	655 (3.11)	874 (3.28)
catabolic process	671 (3.19)	643 (2.41)
electron transport	462 (2.19)	681 (2.56)
lipid metabolic process	586 (2.78)	798 (3)
photosynthesis	140 (0.66)	144 (0.54)
protein metabolic process	2347 (11.15)	4137 (15.53)
secondary metabolic process	461 (2.19)	421 (1.58)
abscission	11 (0.05)	5 (0.02)
embryonic development	536 (2.55)	505 (1.9)
flower development	255 (1.21)	213 (0.8)
ripening	10 (0.05)	3 (0.01)
regulation of gene expression, epigenetic	95 (0.45)	150 (0.56)
reproduction	905 (4.3)	846 (3.18)
response to abiotic stimulus	1238 (5.88)	904 (3.39)
response to biotic stimulus	683 (3.24)	527 (1.98)
response to endogenous stimulus	985 (4.68)	1052 (3.95)
response to external stimulus	441 (2.09)	263 (0.99)
response to stress	1352 (6.42)	1094 (4.11)
transport	1304 (6.19)	1959 (7.36)

To demonstrate the potential of our microarray as an alternative to the existing Citrus GeneChip [19], a comparison between unigenes present in both platforms was performed. First, to equally evaluate the number of genes represented in every chip, we assembled the consensus sequences of the unigenes in the Affymetrix chip according to our assembly parameters (see Materials and Methods). The 33,879 transcripts were reduced to 24,400 unigene clusters (or "superunigenes"), against the 21,000 present in our cDNA array. In addition, we have estimated how many genes are represented in our microarray and not in the Affymetrix one. A BLAST search of the sequences represented in our cDNA array against the consensus sequence of those included in the Affymetrix chip revealed that 6248 genes did not found a positive match with E value lower than 10^-20 ^(7064 with E value lower than 10^-50^) [see Additional file 3]. It implies that they could be analyzed only if using our cDNA array. These results demonstrated that the microarray platform presented in this paper constitutes a complementary tool to the Affymetrix GeneChip for genome-wide transcriptomic analysis in citrus plants.

### Database and website

**Figure 2:**
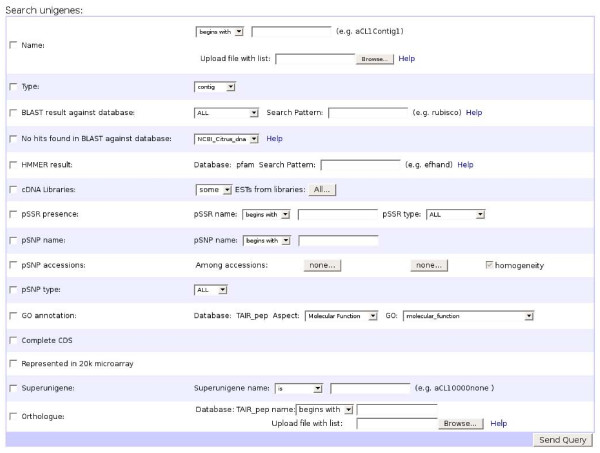
**Database query page**. Unigenes represented in the microarray can be searched by using any combination of structural and functional criteria in the queries.

**Figure 3:**
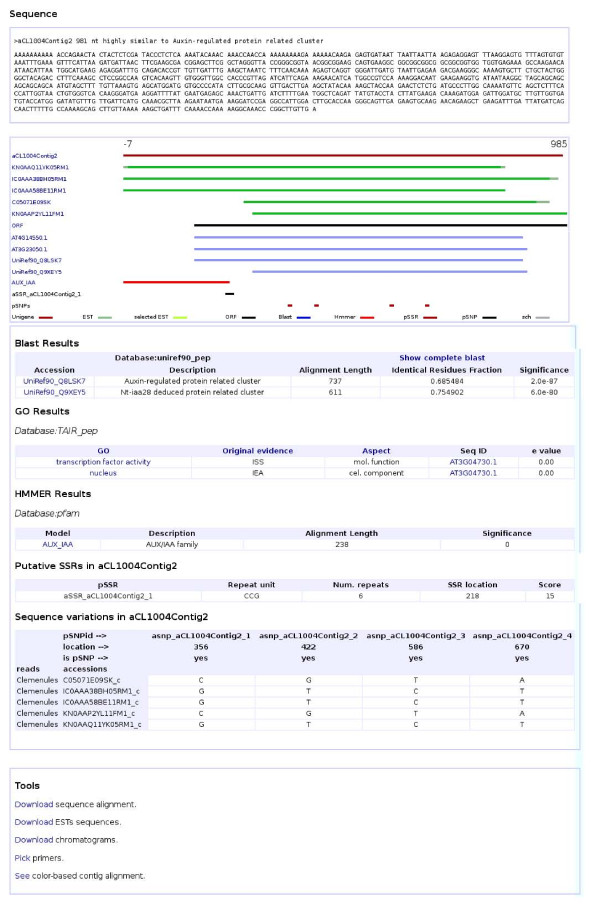
**Individual unigene page**. Individual unigene page shows the results of assembly and annotation for a single unigene. It also offers some links to different tools for data downloads and primer design.

Using the EST2uni package [26], a web-browsable database was created [1] and populated with information about the unigenes represented in the microarray, including cDNA libraries, isolated clones, raw and processed nucleotide and protein sequences, and results of all the structural and functional annotation of the unigenes, like general description, BLAST hits, putative Arabidopsis ortholog, microsatellites, putative SNPs, GO classification [24] and PFAM [25] domains. The web interface to the database is not just a collection of simple tables showing the data, or a simple query to search by using sequence identifiers or keywords. It also allows combination of almost every different functional and structural annotation criteria in the queries (Figure 2). Additionally, bulk queries using a file with a list of unigene names or orthologs are implemented. The unigenes obtained as query results can be inspected individually, but also bulk downloads of the sequences, names or orthologs are allowed. The individual unigene web page view shows graphical and textual summaries of the assembly and annotation processes (Figure 3). Hyperlinks to the first hits of the external databases searched with BLAST are provided, as well as their descriptions and E values. The full BLAST results can also be retrieved. Gene Ontology annotation results are also shown in a table with links to the GO term description pages, using the AmiGO tool [27].

### Functional catalogue of citrus genes expressed in the globular embryo

Embryogenesis is a critical stage of the plant life cycle. The egg cell develops into a multicellular organism via a precise sequence of events [28]. During the first phase of embryogenesis, the body plan is being established, consisting in a shoot meristem, cotyledons, hypocotyl and root meristem along the apical-basal axis, and a concentric arrangement of epidermis, ground tissue and vascular cylinder along the radial axis. Understanding the molecular mechanisms underlying embryogenesis can provide insight into developmental and metabolic regulation of this important stage of plant biology, and a big effort has been made in the two last decades in that direction [29]. A number of important genes and pathways have been identified, and recently, global analysis in Arabidopsis has been performed to identify a set of expressed genes during different stages of early embryogenesis [30].

We have performed a pilot experiment to catalogue the set of expressed genes in the citrus late globular embryo. Citrus exhibit polyembrionic seed development [31]. In many species, non-zygotic embryos develop from the maternal nucellar tissue of the ovule surrounding the sexual embryo sac and develop together with the zygotic one. We crossed *Citrus clementina *(cv. Clemenules) with Fortune (*C. clementina × C. tangerina*) (see Materials and Methods) to obtain monoembryonic seeds and assure the analysis of expression only in zygotic embryos.

First, this experiment constitutes a proof of use of our microarray, demonstrating the utility of a multispecies citrus cDNA microarray for expression studies. Second, Arabidopsis orthologs of many genes present in the microarray are already known to be expressed in the embryo, and it would be interesting to confirm whether these genes are also expressed in citrus embryos. Moreover, the study could reveal novel interesting genes expressed during embryogenesis that initiate future works aimed to decipher their implication in this process.

Five biological replicates were performed. Correlation between replicates ranged between 75% and 90%. A total of 13,341 genes were considered present in the late globular embryo, according to the criteria explained in Materials and Methods [see Additional file 4]. That constitutes the 63% of the 21,081 citrus unigenes examined in our microarray. In a recent paper, [30] found 77% of the 22,800 genes of the ATH1 Arabidopsis Genechip to be expressed in the torpedo stage of embryogenesis. Although the number of present genes should be taken as an estimation depending of the threshold values applied in each case, it reveals that virtually the whole cellular machinery is activated during embryogenesis, reflecting the high metabolic activity of meristematic and differentiating cells.

Although mainly studied in Arabidopsis, overall processes during plant embryogenesis are thought to be similar in other species [32]. Of the 293 EMB genes from Arabidopsis catalogued by the SeedGenes project [33], aimed to identify genes that give seed phenotype when disrupted by mutation, 210 of them had a citrus ortholog and were present in the microarray, and 71% of these were found expressed in the globular embryo of citrus [see Additional file 5]. The remaining ones could be present in a different embryo stage, or not detected due to their low expression [30], although the possibility of not being expressed in the citrus embryo do not has to be neglected. Citrus orthologs of the Arabidopsis genes involved in embryo pattern-formation [34], could also be detected by our microarray: orthologs of GNOM, a gene involved in the establishment of the apical-basal axis, MONOPTEROS, whose mutation alters the normal division of embryonic cells, ZWILLE, involved in establishing the primary shoot meristem in the embryo, or KEULE, gene responsible for the correct cytokinesis of the cell, were also expressed in the citrus embryo.

Other genes or gene families recently known to have a role in plant embryogenesis are also expressed in citrus embryos. Involvement of cell wall and remodelling of cell architecture [35], regulation of mRNA stability and translation through poly-A binding proteins [36,37], regulation of development through pentatricopeptide repeat proteins [38], the involvement of vesicle trafficking in organ development [39] or the role of cell cycle genes in early stages of embryogenesis [40] has been confirmed in citrus embryos by expression of sets of genes belonging to these functional categories. Similarly, the well described role of auxins in establishment of embryo polarity [41] or the recent implication of brassinosteroids in the acquisition of embryonic competence [42] was confirmed in citrus embryos by expression of citrus orthologs of genes related to signalling and biosynthesis of these hormones [see Additional files 6, 7, and 8].

Much less is known about how early embryos prepare themselves for pathogen attack. It has been suggested that developing barley embryos activate a developmental defense activation programme where expression of defence genes is explained to involve control by developmental signals rather than induction by pathogens [43]. Lipoxygenases (LOX1 and LOX2) enzymes, that catalyse the first committed step in JA biosynthesis, have been described to be expressed in developing embryos [44]. We also found expression in globular citrus embryos of LOX1 and LOX2 homologues and of an ortholog to AT1g67460, a 13-lipoxygenase enzyme considered so far to have minimal activity in embryos. Moreover, functional classification of present genes reveals around 9% of genes belonging to the category "response to stress", 8% to the category "response to abiotic stress, 3% to the category "response to abiotic stress", and 3% to the category "Defense". These data point towards a deployment of protection mechanisms in the citrus seed, already activated at the globular stage.

## Conclusion

We have constructed a citrus 20 k cDNA microarray which can be used for gene expression analysis in different species of citrus. We also provide access to a web-browsable database as a companion tool for this microarray. The database contains every structural and functional annotation related to the unigenes represented in the microarray. From a series of experiments on embryos development in citrus, it could be stated that our microarray allows reproducible global expression analysis in citrus, and that citrus embryogenesis share with the model plant Arabidopsis thaliana many aspects of the developmental programme aimed to established the basic body plan of the adult plant. We would like to offer this microarray and the companion database to the citrus research community with the hope that future use of these genomic tools will uncover clues of the transcriptional regulation of genes in different citrus species, and during different aspects of productivity, like plant resistance, plant development, or fruit quality.

## Methods

### Microarray probe selection

ESTs processing and assembly were performed by using EST2uni [26], an open, parallel software package which uses different standard EST analysis tools for automated EST preprocessing, assembly and unigenes annotation. For the present work, EST2uni was used with the following tools. Raw sequences and base confidence scores were obtained from raw chromatogram files using the program phred [45,46]. Low-quality and cloning vector regions were removed from the sequences with Lucy [47], and ESTs that were left with less than 100 non-vector good-quality bases after trimming were discarded from further analyses.

Repetitive elements and low-complexity regions were masked with RepeatMasker [48] and SeqClean [49], respectively. For repeat masking, the eucotyledons-specific repeats database was used. Vector sequence contaminants were also removed with SeqClean, using NCBI's UniVec database [50]. Clean, vector-free EST sequences were submitted to dbEST division of GenBank (accession numbers CX286781 to CX309414, and FC868488 to FC932655). Assembly of reads in contigs and singletons to estimate the redundancy of the ESTs, get the consensus sequences of the redundant ones, and obtain the unigene set was made with tgicl [51], using the following default parameters: 30 bases minimum overlap length, 94% minimum percent identity for overlaps, and 30 bases maximum length of unmatched overhangs. Poly(A/T) tails and open reading frames (ORFs) were predicted for the unigenes using ESTScan [52]. ESTScan was also used to obtain reverse complimentary sequences of the unigenes when necessary.

A number of unigene clusters (or "superunigenes"), grouping different unigenes with extensive sequence overlap (more than 300 bp with more than 90% identity, and covering more than 50% of the length of one of the unigenes), were obtained from the initial unigene set using BLAST. In order to avoid spot cross-hybridization, only one representative per superunigene was selected to be printed in the slides. These representatives were selected according to the following criteria: single PCR product, EST sequence length greater than 300 bp and covering at least 90% of the unigene consensus sequence, and GC content not greater than 80% in a 70 base-long sliding window. Where more than one clone in a superunigene satisfied all the criteria, the longest one was selected to ensure that full-length clones were used for printing when possible. Where no clone in a superunigene satisfied all the criteria, the requirements were progressively relaxed until a representative clone was selected. Only single PCR-product was mandatory, and unigenes without clones satisfying this criteria are not represented in the microarray. The microarray was submitted to the ArrayExpress database (accession number A-MEXP-1017).

### Microarray printing

cDNA clones being the best representative for each superunigene were selected to be PCR-amplified in a final volume of 100 *μ*L using 4 ng of plasmid template, 400 nM of each primer, and 200 *μ*M dNTPs. The reaction products were analyzed by agarose gels, and purified using the Multiscreen-PCR 96-well Filtration System (Millipore). Only PCR reactions yielding single bands were transferred to printing plates, at a final concentration of 150 ng/*μ*L in PRONTO Universal Spotting Buffer (Corning Life Sciences). PCRs were printed onto UltraGAPS aminosilane Corning slides, using a MicroGrid II arrayer (Genomic Solutions). Printed slides were UV-crosslinked at 150 mJ and store in a desiccator until use. Lucidea Universal ScoreCard (GE Healthcare) spike controls were diluted in 100 ng/*μ*L spotting buffer and printed on the array for quality evaluation. Each calibration and negative controls from the Lucidea kit were spotted several times across the whole area of the array. Every selected clone was spotted once.

### Unigene annotation

Using EST2uni [26], structural and functional annotation of unigenes obtained in the assembly step was performed as follows: Di-, tri- and tetra-nucleotide simple sequence repeats (SSR) were detected with Sputnik [53]. Putative single nucleotide polymorphisms (SNPs) were found by EST2uni using a locally developed algorithm. As ESTs have frequent sequencing errors, only positions with a quality score above 39 were considered, and sequence discrepancies between ESTs in the same contig were marked as putative SNPs only if the polymorphism was confirmed by more than one EST in the contig. Lastly, because cDNA libraries were constructed using oligo-dT primer for the reverse transcriptase reaction, unigenes were aligned with the Arabidopsis complete proteins database to predict if there were full-length clones for each unigene.

For the functional annotation of unigenes, BLASTx was carried out in EST2uni against: 1) the UniRef90 non-redundant protein clusters database [22] (downloaded October 2006: UniProtKB release 8.9 of October 2006), and 2) the predicted full set of Arabidopsis thaliana proteins provided by TAIR [23] (downloaded September 2006: TAIR6 of November 2005). BLASTn searches were also made in EST2uni against all the public citrus sequences at GenBank [54], including ESTs (downloaded October 2006). All these analyses were performed using BLAST default parameters and arbitrary non-stringent threshold of 10^-5 ^for E value. Unigenes were annotated with the description of the most similar UniRef90 cluster of proteins. When no significantly similar UniRef90 cluster was found, unigenes were annotated with the first informative description (i.e., not containing words such as "unknown", "anonymous", or "hypothetical") of the BLAST hits, if any, against the databases of Arabidopsis proteins and GenBank citrus DNA sequences, in this order. Unigenes were annotated as highly similar to the first BLAST hit when the E value was lower than 10^-15^. BLASTX hits with an E value higher than 10^-10 ^were not considered for annotation. Gene Ontology [24] annotation of the Arabidopsis more similar proteins was used for GO annotation of the citrus unigenes. A BLASTX E value lower than 10^-20 ^was required to use the GO annotation of the Arabidopsis proteins to the corresponding citrus gene. A HMMER search [55] was also done to identify putative PFAM domains [25] in the unigenes. Finally, a bi-directional BLAST comparison was also performed with Arabidopsis protein database to obtain a set of putative orthologs. In these analyses, two sequences were considered orthologs when each one was the first hit in a BLAST search with the other. All these unigene annotations are automatically stored by EST2uni in a MySQL relational database [56] which can be accessed by Internet using a web browser [1].

### Plant material and RNA extraction

Late globular zygotic embryos were manually extracted from citrus seeds obtained after pollination of *Citrus clementina *(cv. Clemenules) pistils with Fortune (*C. clementina *× *C. tangerina*) pollen, and stored at -80° C prior to use. Five embryos were pooled together and total RNA was extracted using RNAeasy microKit from Qiagen, and quantified using Nanodrop spectrophotometer.

### RNA labeling and hybridization

RNA samples were amplified using MessageAmp II amplification kit from Ambion, using 1.5 g as starting material. 7.5 *μ*g of UTP-aminoallyl-amplified RNA (aRNA) were labeled using Cy3 or Cy5 dye (GE Healthcare), purified using Megaclear columns (Ambion), and quantified using Nanodrop spectrophotometer. 200 pmol of labeled-aRNA were dried and resuspend in hybridization buffer containing 3×SSC, 0.1% SDS, 0.1% salmon sperm DNA and 50% formamide. In each slide, embryo sample was labeled with Cy5. A reference sample was labeled with Cy3 for proper normalization. Microarray hybridization was performed manually using Telechem Hybridization Chambers, following Corning instructions. Briefly, slides were prehybridized for 30 min in 3×SSC, 0.1% SDS, 0,1 mg/mL BSA, rinsed twice with water before drying. Slides were hybridized overnight at 42° C and washed in 2×SSC, 0.1% SDS for 5 min at 42° C, 0.1×SSC, 0.1% SDS for 10 min at room temperature, and 0.1×SSC for 5 min at room temperature. Slides were dried in a table centrifuge and scanned using a GenePix 4000B scanner from Molecular Devices, at 10 *μ*m resolution, 100% laser power and at PMT values adjusted so that total intensity in both channels is equal. Microarray images were analysed using GenePix 6.0 software (Molecular Devices).

### Experimental design and data analysis

Fruits were randomly collected from different field plants. Five biological replicates were done, each one containing five embryos coming from different fruits. Slides were global median normalized so that the median of the median of ratios of every valid spot is equal to 1. After normalization, signal in negative controls was checked to be undetectable, and average signal of internal controls known to be expressed during Arabidopsis embryogenesis was checked to be similar in all replicates. A gene was considered "present" in a microarray if its Cy5 median intensity was above two times the median intensity of its local background. A gene was considered "present" in the embryo if it was considered "present" in at least four of the replicates. Functional interpretation was done with FATIGO+ [57], using the corresponding Arabidopsis ortholog lists.

## Authors' contributions

MAM–G, MCM, JS, and JG generated the PCR set for microarray printing. JJ provided the plant material for microarray hybridizations. MAM–G and JG set up and spotted the microarrays. NM and MAM–G did the microarray hybridizations. JF and JG conceived the design of the microarray, and drafted the manuscript. JF did the bioinformatics work for the EST processing, unigene assembly, clustering and annotation, database population, and website setting up. JG guided and coordinated the design and generation of the PCR sets, the printing of the microarrays, and the hybridizations. All authors read and approved the final manuscript.

## Supplementary Material

Additional file 1 **Functional annotation of the genes represented in the microarray**. The file shows functional annotation of the genes represented in the microarray, including the ID, description and E value of the first BLAST hit from the databases used for annotation (UniRef90 [22] and Arabidopsis TAIR full set of proteins [23]), as well as their Gene Ontology classification [24] and pfam domains [25]. The file is a tab-delimited plain text file with one gene per line. Click here for file

Additional file 2 **Total distribution of citrus and Arabidopsis unigenes along the different GO functional categories**. The file shows the total numbers of citrus and Arabidopsis unigenes belonging to the main Gene Ontology categories, along with the corresponding percentages to the total number of citrus unigenes represented in the microarray and the total number of Arabidopsis genes, respectively. Results are presented independently for the three different GO ontologies. The file is a tab-delimited plain text file using indentation to reflect the hierarchical dependence among GO terms. Click here for file

Additional file 3 **Citrus unigenes considered to be expressed in the late globular embryo**. The file shows the IDs of the citrus unigenes considered to be expressed (see Material and Methods) in the late globular embryo. It is a plain text file with one ID per line. Click here for file

Additional file 4 **Arabidopsis EMB genes which have a putative ortholog expressed in the globular embryo of citrus**. The file shows the IDs of the corresponding Arabidopsis genes. It is a plain text file with one ID per line. Click here for file

Additional file 5 **Biological Process Gene Ontology classification of the Arabidopsis genes orthologs to the unigenes expressed in the late globular embryo of citrus**. The file shows the number, percentage, and IDs of the Arabidopsis genes belonging to each GO category. The classification at GO hierarchical levels 3 to 9 is showed. The file is a tab-delimited plain text file with one GO category per line for each GO level. Click here for file

Additional file 6 **Molecular Function Gene Ontology classification of the Arabidopsis genes orthologs to the unigenes expressed in the late globular embryo of citrus**. The file shows the number, percentage, and IDs of the Arabidopsis genes belonging to each GO category. The classification at GO hierarchical levels 3 to 9 is showed. The file is a tab-delimited plain text file with one GO category per line for each GO level. Click here for file

Additional file 7 **Cellular Component Gene Ontology classification of the Arabidopsis genes orthologs to the unigenes expressed in the late globular embryo of citrus**. The file shows the number, percentage, and IDs of the Arabidopsis genes belonging to each GO category. The classification at GO hierarchical levels 3 to 9 is showed. The file is a tab-delimited plain text file with one GO category per line for each GO level. Click here for file

Additional file 8 **Citrus unigenes included in our 20 k cDNA microarray not represented in the Citrus Affymetrix GeneChip**. The file shows the ID and annotation of the unigenes included in our cDNA array with no BLASTN hit found below an E value threshold of 10^-50^among the consensus sequences of the unigenes represented in the Citrus Affymetrix GeneChip. The corresponding E value of the first hit found is also indicated. The file is a tab-delimited plain text file with one citrus unigene per line. Click here for file
